# Research on the Flow Parameters of Waste Motion in a Rotary Kiln with the Use of the Tracer Method

**DOI:** 10.3390/s23146526

**Published:** 2023-07-19

**Authors:** Tomasz Jaworski, Agata Wajda

**Affiliations:** 1 Department of Technologies and Installations for Waste Management, Silesian University of Technology, Konarskiego 18, 44-100 Gliwice, Poland; tomasz.jaworski@polsl.pl; 2 Institute of Energy and Fuel Processing Technology, Zamkowa 1, 41-803 Zabrze, Poland

**Keywords:** rotary kiln, flow parameters, tracer method, signal-response experiment, algorithm for residence time, residence time distribution, sensor method, dispersion

## Abstract

The motion of input material in a rotary kiln is an important aspect of its operation. This can be observed especially in the case of the implementation of the hazardous waste incineration process in this device. The values of the flow parameters, mainly the residence time and the degree of mixing, can determine the proper and safe treatment of waste. The relationships that occur in the layer of solid material in a rotary kiln have not been completely recognized. This article presents a research method that involves an experiment on a laboratory stand simulating a rotary kiln in association with a dedicated algorithm. Multi-criteria tests were carried out. The adopted research method was the tracer method. It used a tracer which, subject to the same transport conditions as other material particles, provided information on the characteristic of the motion of tested materials in the rotating cylinder. The application based on the residence time distribution (RTD) algorithm returned information about the characteristics of the motion of the material in the rotary cylinder in terms of residence time distribution and the degree of mixing. This tracer method, together with stimulus impulses on the grate and a dedicated RTD algorithm, was used here as a sensor method to examine the characteristics of material motion on various grate systems. The data obtained as a result of this research may include, among others, the boundary conditions for numerical simulations of processes carried out in a rotary kiln.

## 1. Introduction

A rotary kiln in waste management was used to perform the thermal transformation of hazardous waste in waste incineration plants. The co-incineration of waste in cement kilns to produce a clinker can also be considered in terms of using the rotary kiln [1,2,3,4]. It was estimated that about 60 kg of hazardous waste per person was generated annually, which translated into a considerable amount of 480 million Mg/year [5]. This is a very diverse group of waste that can be recovered; however, some types should be incinerated with strict process parameters. This applies primarily to medical waste. Despite the currently observed phenomenon of reducing thermal treatment methods in waste management, related, among others, to the introduction of the idea of a circular economy [6,7], for some hazardous waste, there are currently no methods that allow for their safe management other than incineration.

A significant prerequisite in the description of high-temperature processes in combustion chambers is the description of the motion and forces of each element present in the reaction system. They are flow parameters, among which the most important are the residence time of the material in the chamber and the degree of material mixing [8,9,10]. They directly affect the efficiency of the thermal process, as well as the quality of its products. This is particularly important in the process of hazardous waste incineration, as post-process waste should not be harmful. This effect is achieved when the process is carried out correctly, which is largely influenced by the residence time of the waste in the reactor and the degree of its mixing [11,12,13]. An analysis of the essential characteristics of the material motion could be performed by adapting the universal theory of flows described, among others, in [14,15]. Within this theory, two cases of flow could be distinguished: perfect mixing and piston flow. In a rotary kiln, there was a dispersion flow of an unspecified nature, which differed to a greater or lesser extent from the state of perfect mixing and the plug flow (no mixing).

In the case of studying the movement of the input material in a rotary kiln, the literature most often indicates a theoretical or empirical approach. The most important theories of material mass transport in a rotating cylinder include the theories of Seamann and Danckwerts [16,17]. Subsequent models of material movement in a rotary kiln are based on these relationships [18,19,20,21,22]. This empirical approach is presented, among others, on [23], where the residence time of solid particles in a rotary kiln was measured, and the factors influencing the average residence time and residence time distribution, such as rotational speed, inclination, feed speed and particle size, were evaluated.

Sensors, together with algorithms, are widely used in technological processes, where they serve to synchronize and stabilize the system [24]. They are used in numerous branches of the economy, including unmanned aerial vehicles (UAV), robotics for the implementation of circuits, the modeling of heat conduction, and many other applications [25,26]. In the case of the rotary kiln operation, considerations generally relate to the study of phenomena other than the movement of a material bed. Among the algorithms dedicated to thermal processes carried out in a rotary kiln, research can be mentioned [11,27,28]. The authors [11] present an application to optimize the work of operators of hazardous waste incineration plants and manage the stream of the hazardous waste fed. In [27], an approach based on combining the moving window and multi-channel convolutional neural networks (MWMC-CNN) was proposed to simulate the use of electricity and coal in the cement production process. In [28], the use of wireless sensor networks (WSN) was considered in the context of optimizing the operation of a cement plant.

In order to study the flow conditions in the rotary reactor, this study proposes a research methodology combining the tracer method and a dedicated algorithm. It can be defined as a sensor method, as it envisages stimulating the grate with a batch of markers, tracking its movement in the device, and then receiving data—material movement parameters. These data are then entered into a program based on the RTD (Residence Time Distribution) algorithm. On the basis of the conducted tests, important parameters of the material motion description could be determined for the considered cases, including the residence time distribution (RTD) and the Peclet number (Pe_L_). The residence time distribution method in many analyses in the area of high-temperature processes was characterized by a relatively small computational effort in relation to the model using the laws of continuity. The Peclet number is a convenient parameter for flow dynamics in reactors, including a rotary kiln. In general, in numerical simulations of rotary kiln systems, it is common to simplify the bed transformations of solid material while at the same time meticulously modeling the gas phase. Meanwhile, the behavior of solid material particles during thermal transformations is very important, especially in the context of hazardous waste incineration plants. Once complete, the thermal transformation of waste allows for the neutralization of harmful substances contained therein. Therefore, efforts should be made to ensure that the mass transport of the material in the rotary kiln is not simplified to such a large extent. It provides information that can increase the accuracy of the modeling thermal processes. Hence, it is important to obtain input data for numerical simulations, especially those characterizing the behavior of the bed of solid material.

## 2. Research on the Flow Parameters Using the Tracer Method

Studying the properties of the motion of solid material in a rotating cylinder was carried out on a designed laboratory stand using the tracer method. The subject of this study included selected types of solid materials with different rheological properties. The test variants were defined. For each set of parameters, the tests are carried out in at least three repetitions on a stand that simulated the operation of a rotary kiln in a hazardous waste incineration plant, not taking into account the thermal process (“cold tests”).

### 2.1. Properties of the Tested Materials

Five types of solid materials were selected for the tests: pellet, wood bark, LECA (Lightweight Expanded Clay Aggregate), wood chips, and a mixture of wood bark and expanded clay in a 1:1 mass ratio. These were characterized by diversification in terms of rheological properties. They were selected in such a way that they corresponded to the substrates and products of the actual process of the incineration of solid waste in a rotary kiln. Therefore, some of them referred to selected types of waste transformed in a rotary kiln (pellet, wood bark, wood chips), including a mixture of wood bark and expanded clay, to form these wastes during transformation, where expanded clay was the equivalent of combustion products, i.e., ashes and slags furnace. Table 1 summarizes the rheological characteristics of the selected materials.

### 2.2. The Laboratory Stand

The laboratory stand is the equivalent of a rotary kiln. The construction of this device can be described as an elongated, cylindrical steel drum of a specific diameter. Most often, it works in a high-temperature regime; therefore, its interior is lined with a refractory layer. The kiln rotates around its own axis with a rotational speed in the range of 0.25–4.5 revolutions/minute. Its protection against the improper movement of the charge is the inclination of the kiln to the ground at a slight angle. This forces an unambiguous direction of waste mass transport in this device [29,30].

The basic element of the laboratory stand, its scaffolding, included a set of parallel cylinders mounted onto a frame. These were driven into rotation by the drive system. The drive consisted of an electric autotransformer, a motor, a pulley, and racks with chains mounted on rods, which were fixed inside the cylinders. On this structure, between two cylinders, a PVC cylinder was placed. The dimensions of the cylinder were selected to reflect industrial rotary kilns. The comparative criterion here was the ratio of the length of the cylinder (L) to its internal diameter (d), which, based on the study [31], was in the range of 2.63–6.43, which was, on average, 4.53. For the selected cylinder, the L/d ratio was 4.47. The cylinder ends were different. The outlet end was not restricted, while the inlet end was flanged to prevent the material from escaping. There was an opening in the flange with a diameter slightly larger than the diameter of the second PVC cylinder, which was flush with the surface of the flange. The second pipe was equivalent to the material feeder. This element was mounted on a stand, thanks to which it did not repose against the flange at the cylinder inlet. The material was fed into the rotating cylinder by means of a piston.

Parameters of the cylinder:

- Length: L = 0.85 m,
- Internal diameter: d = 0.19 m,
- Length/internal diameter: L/d = 4.47,
- Cross-sectional area: *p* = 0.028 m^2^.

The performed tests were carried out without the use of thermal processes. During the tests, there was no heat or mass exchange, and only the physical properties were tested.

**Figure 1:**
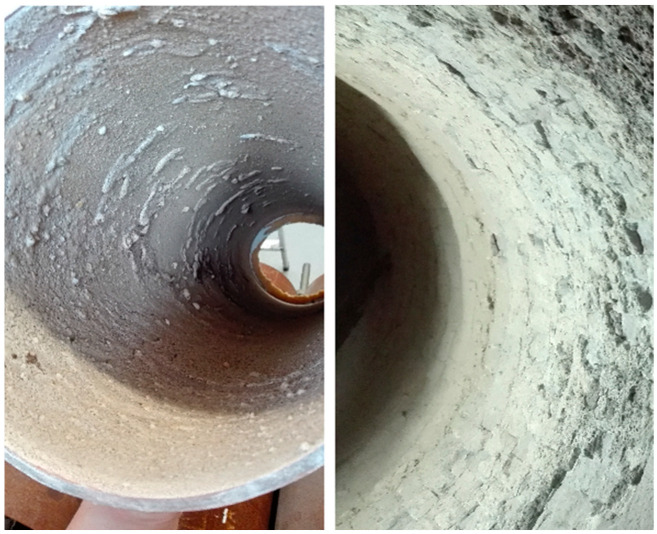
The inner layer of the rotary cylinder and the rotary kiln.

The inside layer of the cylinder was covered with a bonding layer and sand, which imitated a layer of the lining in the rotary kiln. A comparison of the interior of the rotary cylinder from the laboratory stand with the interior of the rotary kiln on a real-scale installation is shown in Figure 1.

### 2.3. The Tracer Method

**Figure 2:**
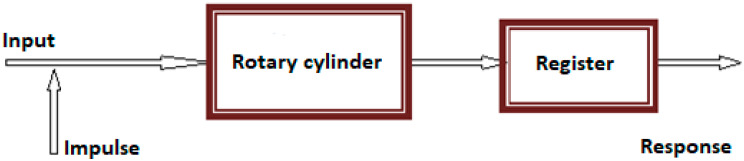
Scheme of the impulse-response experimental setup.

In order to determine the characteristics of the bed of solid material in a rotating cylinder, the tracer method (also known as the impulse method or impulse-response method) was used. This method used an indicator substance, called a marker or tracer, which acted as a signal. It was introduced into the tested system at a specific time and at a specific amount in a place identical to the place where the tested material was dosed. Figure 2 shows the basic idea of carrying out the research using the impulse method.

**Figure 3:**
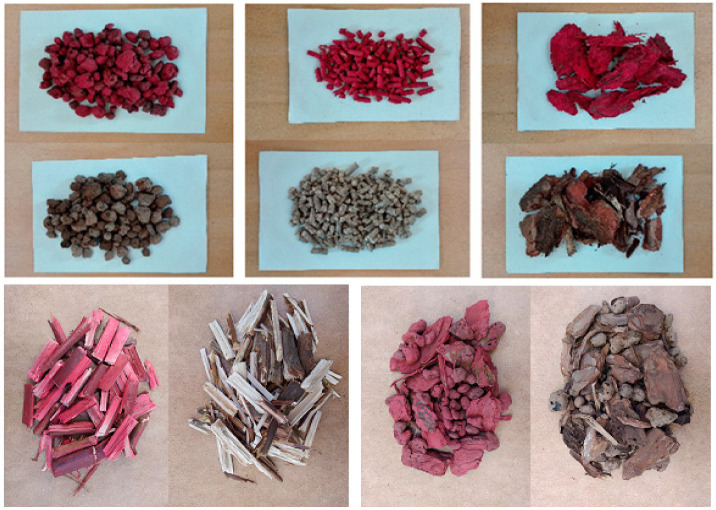
Tested materials and their markers.

The indicator was assumed to mix with the base material and travel the same distance under the same flow conditions. Hence, by examining the behavior of the tracer, it was possible to determine the characteristics of the tested material [15,32]. The markers in the test were prepared from basic materials that had been colored for easy separation. Recording the concentration of this substance as a function of time at the outlet from the rotary cylinder allowed us to determine the residence time distribution function. The tested materials and their markers are shown in Figure 3.

The proper selection and introduction of a tracer to the system was an important element of this study. The tracer should have several features:

- Physical and chemical properties that are the same or similar to the tested material,
- No interaction with the tested material and no influence on its flow,
- The ability to distinguish the tracer from the base material in the test [15,33].

### 2.4. The Course of the Tests

The scope of the multi-variant tests carried out included the selection of three process variables and their values, as well as an analysis of the impact of the changed parameters on the material flow in the rotary cylinder. The following variables were selected:

- The cylinder rotational speed,
- The angle of inclination to the ground,
- The type of feed material (variation in rheological properties).

Table 2 lists the values and the process variables for individual test variants.

The total stream of the input material corresponded to the cylinder filling factor, which was assumed to be at a level of 15%. The share of the tracer in the mass stream of the tested material was 10%. The material prepared in this way was divided into equal portions, where the tracer was a portion intended for one batch. A laboratory stand was prepared for specific test variants.

The course of each trial was as follows:

- The introduction of batches of the material in order to reach a steady state,
- The introduction of two batches of material,
- The introduction of the tracer batch,
- The batch of material was continuously introduced until the lack of presence of the tracer in the received material.

The input and output of the material occurred at predetermined time intervals. The test began with obtaining a steady state in the working cylinder, which could be defined as similar values of the stream supplied to the stream received. One portion of the tracer was then fed, continuously collecting material at predetermined time intervals. After the test, the received batches of material were weighed their division into plain and colored material. As the research concerned, the residence time and the tracer had a representative function; here, the share of it in a given portion of the received material was checked in relation to the total mass of the tracer and introduced into the cylinder as part of the impulse.

## 3. Algorithm for Determining the Residence Time Distribution

### 3.1. Introduction to the Algorithm

The actual residence time of the material particles in the rotary kiln and the degree of their mixing were important parameters from the point of view of motion description, as well as determining the possibility of optimizing this issue. There have been different approaches to this issue in the literature. The analysis of the essential characteristics of the movement of the material in the kiln could be performed by adapting the universal theory of flows described, among others, in [14,15]. Within this theory, two cases of flow were distinguished with perfect mixing and piston flow.

In a rotary kiln, we dealt with the dispersion flow of an unspecified nature, which differed to a greater or lesser extent from ideal models. For practical reasons, it is important to assess the degree of approximation to the ideal state. The criterion for such an assessment was provided by two distribution functions of the real residence time of particles in the rotary kiln:

- *E*(*t*) represents specifying the molar (mass) fraction of particles with a residence time and within a certain range in the stream leaving the device,
- *F*(*t*) represents the distribution function of the residence time distribution, also called the residence time distribution.

Both functions are the criterion of the previously mentioned compliance of the calculation results with the practical data. Residence time distribution curves were the only and objective picture of the flow dynamics in real conditions.

For the residence time distribution function defined in this way, the following was applied:

$$\int_{0}^{\infty }E\left(t\right)dt=1$$

The value of the function *F*(*t*) for time *t* provided the part of the stream of waste particles leaving the rotary kiln with residence time in a range from 0 to *t*. The relationship between these two functions, resulting from the definition, could be calculated as follows:

$$F(t)=\int_{0}^{t}E\left(t\right)dt$$

It follows that:

$$\frac{dF(t)}{dt}=E(t)$$

Moments of these distributions were very helpful in characterizing and comparing the residence time distributions. Typically, we used a first moment and a second central moment, called the variance, to evaluate the residence time distribution.

The first moment equaled the average residence time:

$$\bar{t}=\int_{0}^{\infty }tE\left(t\right)dt$$

The variance characterized the dispersion of the residence time relative to the mean value:

$$\sigma_{t}^{2}=\int_{0}^{\infty }{\left(t-\bar{t}\right)}^{2}E\left(t\right)dt=\int_{0}^{\infty }t^{2}E\left(t\right)dt-{\bar{t}}^{2}$$

The first and second moments of the random variable *t* uniquely characterized the residence time distribution. In practice, they were used as convenient criteria for comparing the distribution curves. For comparative purposes, it was more advantageous to use dimensionless time in relation to the average residence time of the material in the rotary kiln:

$$\theta =\frac{t}{\bar{t}}$$

This parameter is called the relative residence time. It is also a random variable with the same distribution functions as *t*.

F(θ)=F(t)

$$E(\theta )=\bar{t}E(t)$$

Using dimensionless time, the moments and width of the distribution curve could be represented as follows:

$$\bar{t}=\int_{0}^{\infty }\theta E\left(\theta \right)d\theta =1$$

$$\sigma_{\theta }^{2}=\frac{\sigma_{t}^{2}}{{\bar{t}}^{2}}=\int_{0}^{\infty }{\left(\theta -1\right)}^{2}E\left(\theta \right)d\theta =\int_{0}^{\infty }\theta^{2}E\left(\theta \right)d\theta -1$$

An important issue here was the interpretation of the response curves in real reactors and their connection with the Peclet number given by the formula:

$${Pe}_{L}=\frac{uL}{D_{L}}$$

where:

*L*—reactor length, [m],

*u*—linear speed, [m/s],

$D_{L}$—longitudinal dispersion coefficient, [m^2^/s].

The $D_{L}$ coefficient is an effective substitute value (constant in the cross-section), which allowed us to capture the mixing phenomena along the reactor axis in a uniform manner, regardless of their nature.

The value of ${Pe}_{L}$ characterized the flow dynamics and was a measure of the intensity of longitudinal mixing in the reactor. The shape of the residence time distribution curves *E*(*t*) and *F*(*t*) depended on its value. For borderline cases, it took the value:

- ${Pe}_{L}$ = 0 ($D_{L}$ = ∞)—perfect mixing (tank reactor),
- ${Pe}_{L}$*=* ∞ ($D_{L}$ *=* 0)—no mixing (tube reactor).

A flow in which the mixing phenomenon can be described by the number ${Pe}_{L}$ or the coefficient $D_{L}$ and where there is no variation in the concentrations in the cross-section of the stream is called dispersion flow.

Depending on the assumed boundary conditions in the considered case, various models of the dispersion flow were obtained, binding the distribution functions *E*(*θ*) or *F*(*θ*) and their parameters $\sigma_{\theta }^{2}$, $\bar{\theta }$ with the Peclet number. The theoretically determined dependencies of variance on the Peclet number were of the greatest importance. If the variance $\sigma_{t}^{2}$ or $\sigma_{\theta }^{2}$ was determined from the experimental curves *E*(*θ*) before using the appropriate equation of boundary conditions, it was possible to estimate the value of the number ${Pe}_{L}$. The Peclet number found in this way provided a measure of the average dispersion in the working zone of the device (between the signal input and output points).

With regard to the combustion chamber of the rotary kiln, in which the flow was dispersive *D*/*uL* > 0.01, and for the boundary conditions of the “open vessel”, the residence time spectrum in the mathematical notation could be expressed by the equation:

$$E(t)=\frac{u}{\sqrt{4\pi tD}}exp\left[-\frac{(L-ut{)}^{2}}{4tD}\right]$$

or in the dimensionless form:

$$E(\Theta )=\frac{1}{\sqrt{\left(4\pi \Theta {Pe}_{L}^{-1}\right)}}exp\left[-\frac{(1-\Theta {)}^{2}}{4\Theta {Pe}_{L}^{-1}}\right]$$

On the basis of the above general relationships, an algorithm dedicated to determining the residence time distribution (RTD) and the Peclet number was developed.

### 3.2. Algorithm for the RTD Calculation

Bearing in mind the lack of knowledge of the values that allowed the calculation of the ${Pe}_{L}$, the analytical form of the above equation could be presented (by introducing independent parameters: “*α*” *i* “*b*”) in the form:

$$E(\Theta )=\alpha \Theta^{-0.5}exp\left[b\frac{(1-\Theta {)}^{2}}{\Theta }\right]$$

This regression function was used to develop the results of the measurement of the residence time distribution and was carried out on the stand for cold tests of the rotary kiln. In this case, to estimate the structural parameters of the regression curve, as reported in [33,34], the least squares method could be used if the function was reduced to the following form:

$$\ln\;E(\Theta )=\ln\;\alpha -0.5\ln\;\Theta +b\frac{(1-\Theta {)}^{2}}{\Theta }$$

In addition, by introducing simplifying notations:

$$z=\ln\;E\left(\Theta \right)$$

x=ln Θ

$$y=\frac{(1-\Theta {)}^{2}}{\Theta }$$

z=α−0.5x+by

Estimating the structural parameters of the regression function was performed by finding the minimum expression:

$$W=\sum (z_{i}-\ln\;M_{i}{)}^{2}$$

$$W=\sum (\alpha -0.5x_{i}+by_{i}-\ln\;M_{i}{)}^{2}$$

For data from the “*m*” sample, the values where ($M_{i},\;\Theta_{i}$), *i* = 1, …, *m*, where: *m*—number of measurements, $M_{i}$—a relative mass of the received tracer (calculated relative to the initial mass of the inserted tracer $m_{0}$), as expressed by the equation:

$$M_{i}=\frac{m_{i}}{m_{0}}$$

Since the equation was a function of two variables *α i b*, the issue came down to finding the minimum of a quadratic function of two variables. A necessary condition for the existence of an extremum was the zeroing of partial derivatives.

$$\frac{\delta W}{\delta \alpha }=0$$

$$\frac{\delta W}{\delta b}=0$$

As a result of the differentiation of the function defined through the equation by variables *α* and *b,* a system of two linear equations was obtained. Since time here was dimensionless with respect to the average residence time $t_{m}$, it was necessary to find a third equation that was a function of only three parameters: *α*, *b,* and oraz $t_{m}$. This was achieved by assuming an additional condition that determined how the solution could be obtained, thus:

- Variant 1: Assumed that the sum of the relative measured masses $M_{i}$ was equal to the sum of the values determined by the regression function *E*($\Theta_{i}$), as expressed by the following equation:
  $$\sum M_{i}=\sum E(\Theta_{i})\mathrm{for}\;i=1,\;\ldots ,\;m$$
- Variant 2: Assumed that the sum of the values determined by the regression function *E*($\Theta_{j}$) was a consequence of the relative notation: mass of the received tracer and residence time. Therefore, this algorithm took into account the following condition:
  $$E(\Theta_{j})=1\mathrm{for}\;j=1,\;\ldots ,\;n$$
  where *n*—the number of values of *E*(*θ_j_*) determined by the regression function.
- Variant 3: The combination of the conditions presented above was the assumption included in the equation:

$$\sum M_{i}=\sum E(\Theta_{i})\mathrm{for}\;i=1,\;\ldots ,\;m$$

$$E(\Theta_{j})=1\mathrm{for}\;j=1,\;\ldots ,\;n$$

Calculations of numerical values of unknown parameters (*α*, *b*, and oraz $t_{m}$) were made by the program taking into account the following three conditions:

$$\frac{\delta W}{\delta \alpha }=0\Rightarrow t_{m}$$

$$\frac{\delta W}{\delta b}=0\Rightarrow b$$

and

- for the variant 1 ⇒ *α* (*a*)
  $$a=\frac{\sum M_{i}}{\sum \Theta_{i}^{-0.5}e^{by_{i}}}$$
- for the variant 2 ⇒ *α* (*a*)
  $$a=\frac{1}{\sum \Theta_{i}^{-0.5}+e^{by_{i}}}$$
- for the variant 3 ⇒ *α* (*a*)
  $$a=\frac{1+\sum M_{i}}{\sum \Theta_{i}^{-0.5}e^{by_{i}}+\sum \Theta_{i}^{-0.5}e^{by_{i}}}$$

To calculate the value of the average residence time of these materials in the rotating cylinder, the computer program used the iteration method, for which it set parameters for the maximum number of iterations and the maximum change to 500 and 10^−6^, respectively. This made it possible to achieve the high accuracy of the solution generated by the program in a relatively short time.

The expression of the dispersion of the residence time of the material in the rotary cylinder in relation to its average value was the variance. In the case of an impulse stimulus, the formula in which the variance was defined is given below:

$$\sigma_{t}^{2}=\frac{\sum t_{i}^{2}E(t_{i})∆t_{i}}{\sum E(t_{i})∆t_{i}}+t_{m}^{2}$$

Additionally, considering that for an open system, the equality was true:

$$\frac{\sigma_{t}^{2}}{t_{m}^{2}}=\frac{2}{{Pe}_{L}^{2}}({Pe}_{L}+4)$$

The value of the Peclet number could be determined, which, being a description of the degree of mixing intensity, classified the tested flow as a flow with low, medium, or high mixing and thus could decide on the correctness of the calculations. If the condition ${Pe}_{L}^{-1}$ > 0.01 was not met (this happened when the mixing of the material in the rotary kiln was small), the average residence time of the material in the kiln could be calculated, according to Levenspiel in [35], straight from the equation:

$$t_{m}=\frac{\sum t_{i}c_{i}∆t_{i}}{\sum c_{i}∆t_{i}}$$

where:

$t_{i}$—the time from when the tracer was inserted to when it was received, [s],

$∆t_{i}$—the time interval for the collection of individual portions of the material, [s],

$c_{i}$—tracer concentration (the share of the tracer weight in a given portion to the weight of the entire tracer), [kg/kg].

The determination of the diffusion value of the Peclet number was (in the case of a large mixing of the material in the furnace) the last operation performed by the developed algorithm.

## 4. Results and Discussion

### 4.1. Results of the Experiment with the Use of Tracer Method

All the listed materials were tested. There were 30 variations in the test plan, and each was performed in three iterations. The results entered into the program and became the basis for considerations constituted with the arithmetic mean of the trials. Table 3 presents exemplary results based on specific test variants.

Analyzing the results of the experiment contained in Table 3, one could notice the influence of the properties of the materials on their residence time in the rotary cylinder. These results are presented for three selected types of materials, which were subjected to the same test conditions. LECA had the longest residence time, followed by the mixture, and the shortest was the wood chips. This suggests the existence of a difference in the residence time of the charge material at various stages of the thermal process. The other two biomass materials simulating raw waste (pellet, wood bark) also showed this tendency in the variant.

### 4.2. The Results Obtained from the RTD Algorithm

**Figure 4:**
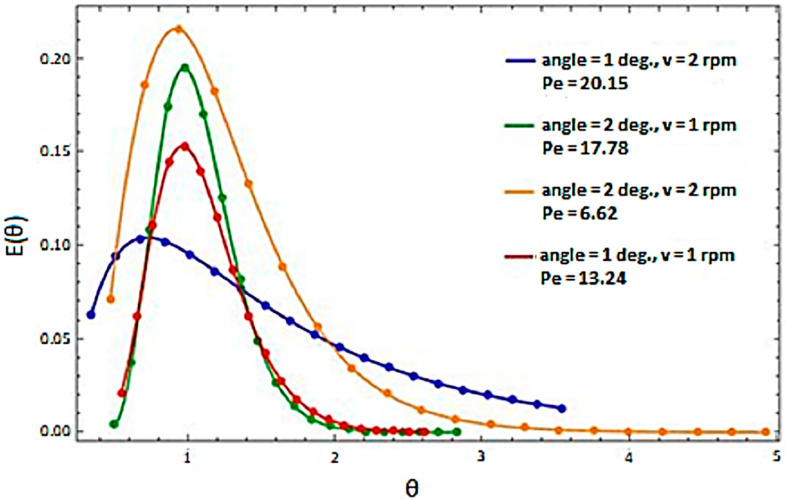
Distribution of residence time for LECA depending on the test variant.

**Figure 5:**
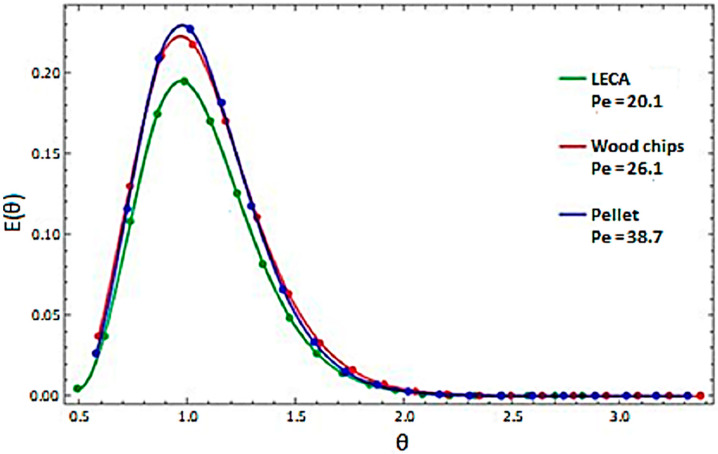
Distribution of residence time for selected materials in the variant v = 1 rpm, angle = 2°.

In order to determine the flow parameters of the material in the rotary cylinder, the data obtained from the impulse method were introduced into the calculation program in accordance with the algorithm presented above. This program returned information, among others, regarding the intensity of longitudinal mixing in the material, which was expressed by the Peclet number. The residence time distributions for selected test variants are shown in Figure 4 and Figure 5. Representative examples of RTD are shown in two approaches:

- A list of four different test variants for the selected material (Figure 4),
- A list of three different materials for a given test variant (Figure 5).

**Figure 6:**
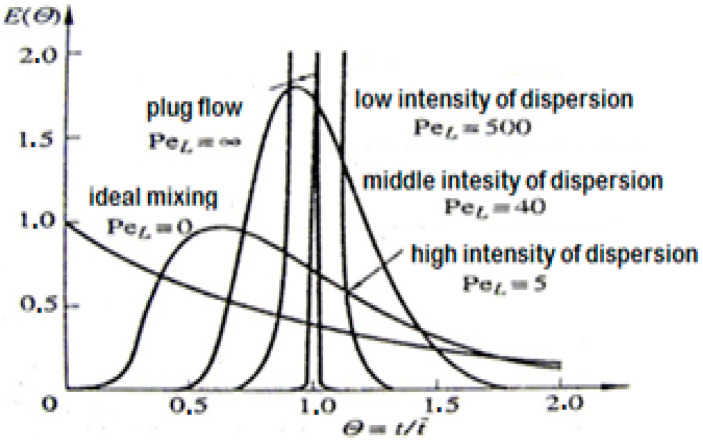
Residence time distribution curves for different dispersions.

As part of the initial analysis, the values of the Peclet number could be compared with the standard curves, which characterized the areas of mixing intensity according to the graph shown in Figure 6.

LECA, depending on the work variants, showed a varied distribution in the residence time, despite the fact that the value of the Peclet number was at a similar level. On the basis of these values and in relation to the chart presented in Figure 6, the type of movement could be generally defined as within the range of the medium dispersion intensity (*Pe_L_* = 40) to high dispersion intensity (*Pe_L_* = 5). Based on [34] and comparing the obtained values of the Peclet number, it could be assessed that the movement of the material in the rotary kiln was, to some extent, similar to the movement of the input material on grate structures found primarily in municipal waste incineration plants.

By analyzing the behavior of individual types of material for a given test variant (Figure 5), it was possible to determine the similarity of the residence time distribution in the rotary cylinder. What distinguished these materials was the intensity of motion, which was the lowest in the case of expanded clay. Analyzing the obtained results, it could be concluded that the Peclet number, followed by the dispersion intensity, depended on the value angle of repose. In this case, the relationship was proportional. However, it should be stated that the material’s motion depended on many factors.

### 4.3. Linear Regression

**Figure 7:**
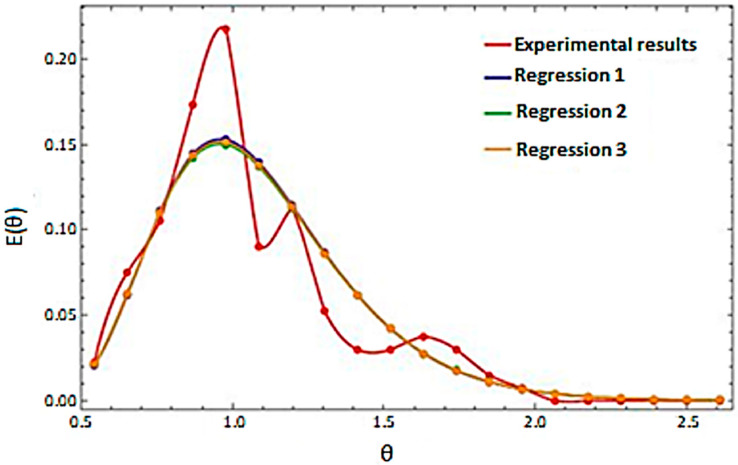
Distribution of residence time for measurements in the variant: LECA, angle = 1°, v = 1 rpm, together with three different regressions performed for this case.

As part of the quality assessment of the program where linear regression was performed. The results of the regression are presented in Figure 7.

The performed regressions were very close to each other. In order to accurately compare the obtained regression results, Table 4 lists the parameters that characterized the motion of the material, such as the mean residence time *t_m_*, Peclet number *Pe_L_*, the standard deviation *ϭ*, for the measurement results, and three performed regressions. These results are presented for the test variant in which the cylinder inclination angle was 1°, and the rotational speed was 1 rpm.

The data contained in Table 4 indicate the convergence of the regression results. Comparing the results obtained using the program with the experimentally determined parameters, slight differences were noticed. This may have resulted from the fact that the algorithmic equations did not take into account the difference in the size of individual grains of the material. The program was sensitive to such changes, although the differences were small. In the case of this comparison, the convergence was most evident for the mean residence time and the Peclet number. This indicates the high quality of the program.

## 5. Conclusions

Two types of conclusions can be drawn on the basis of the conducted research. They concern the dependencies observed during the study on the characteristics of the movement of the material in the rotary cylinder, as well as the quality of the developed program and the possibility of its use.

As a result of the conducted research, certain tendencies were noted. This could be a starting point for the organization of subsequent studies on the motion characteristics in a rotating cylinder. An important general conclusion was the observation concerning the residence time of a given material in a rotating cylinder, which depended to a greater extent on the rotational speed than on the angle of inclination of the cylinder to the ground. The importance of the rotational speed parameter was also emphasized by the characteristics of the residence time of the materials in the rotating cylinder depending on its value (1 rpm or 2 rpm). This was a general trend. For the tested materials, it could be seen that at a rotational speed equal to 1 rpm, the residence of all materials in the rotary cylinder was characterized by greater homogeneity over time—the graph of the distribution function was “flattened”. It was not affected by the angle of the kiln inclination, which was related to the first observation. Another dependence that was revealed in the experiment was the variant with the shortest residence time. For all tested materials, it was a variant with a rotational speed of 2 rpm and an angle of inclination in the roller to the ground of 2 degrees.

In the case of the evaluation of the program that was dedicated to determining the flow parameters, the obtained results of linear regression indicated the high quality of the generated solution. Comparing the values obtained from the program with the values obtained from the experiment, a slight difference was found, but at an acceptable level. This was due, among others, to the fact that the algorithm did not consider differences in the particle size of the tested material. Nevertheless, the program, in a way that reflected reality, allowed us to determine the characteristics of the movement of the input material in the rotary cylinder, primarily in terms of the parameters: residence time and degree of mixing. This is one of the most important factors in determining the safety of the hazardous waste incineration process in a rotary kiln. In addition, the obtained results could be used as input data for modeling the thermal treatment of waste in a rotary kiln.

## Figures and Tables

**Table 1:** Selected properties of the tested materials.

Parameter	Unit	Pellet	LECA	Wood Bark	Wood Chips	Wood Bark + Leca
Bulk density	kg/m^3^	610	278	147	209	198
Apparent density	kg/m^3^	978	664	216	620	345
Porosity	-	0.38	0.55	0.32	0.66	0.43
Angle of repose	°	45	34	27	42	30
Size	mm	20 × 6	8–16	-	30 × 5	-

**Table 2:** Values of process parameters for individual test variants.

Material	Rotational Speed [rpm]	Angle of Inclination [°]
Pellet	1; 1.5; 2	1; 2; 3
LECA	1; 1.5; 2	1; 2; 3
Wood bark	1; 1.5; 2	1; 2; 3
Wood chips	1; 1.5; 2	1; 2; 3
Wood bark + LECA	1; 1.5; 2	1; 2; 3

**Table 3:** The results of the material movement test for the selected variants.

**Variant: LECA, Angle 3°, v = 1 rpm**							
**Time** **[min]**	**Tracer Mass** **[g]**	**Material Mass** **[g]**	**Tracer Share** **[%]**	**Time** **[min]**	**Tracer Mass** **[g]**	**Material Mass [g]**	**Tracer Share [%]**
1	0	290	0	10	14	258	5.26
2	0	288	0	11	18	250	6.77
3	0	284	0	12	14	262	5.26
4	4	278	1.50	13	10	276	3.76
5	12	272	4.51	14	8	278	3.01
6	52	212	19.55	15	6	274	2.26
7	60	202	22.56	16	6	278	2.26
8	26	232	9.77	17	2	282	0.75
9	34	214	12.78	18	0	286	0
**Variant: Mixture, Angle 3°, v = 1 rpm**							
**Time** **[min]**	**Tracer Mass** **[g]**	**Material Mass** **[g]**	**Tracer Share** **[%]**	**Time** **[min]**	**Tracer Mass** **[g]**	**Material Mass [g]**	**Tracer Share [%]**
1	0	252	0	10	28	234	10.85
2	0	256	0	11	20	236	7.75
3	0	256	0	12	12	242	4.65
4	2	252	0.77	13	10	246	3.88
5	18	236	6.98	14	6	248	2.33
6	26	230	10.08	15	6	250	2.33
7	26	228	10.08	16	4	252	1.54
8	52	202	20.16	17	0	254	0
9	48	208	18.60				
**Variant: Wood Chips, Angle 3°, v = 1 rpm**							
**Time** **[min]**	**Tracer Mass** **[g]**	**Material Mass** **[g]**	**Tracer Share** **[%]**	**Time** **[min]**	**Tracer Mass** **[g]**	**Material Mass [g]**	**Tracer Share [%]**
1	0	246	0	9	28	218	11.48
2	0	244	0	10	16	226	6.56
3	0	246	0	11	8	236	3.28
4	0	248	0	12	0	242	0
5	6	240	2.46	13	6	238	2.46
6	30	212	12.30	14	0	244	0
7	90	156	36.89	15	2	242	0.82
8	58	186	23.77	16	0	250	0

**Table 4:** Motion parameters for the measurement results, including regressions for the variant: LECA, angle = 1°, v = 1 rpm.

Data	*t_śr_*, min	*Pe_L_*, -	*Ϭ*, *-*
Experiment results	9.67	13.74	2.81
Regression 1	9.20	13.24	4.08
Regression 2	9.19	13.09	4.10
Regression 3	9.20	13.17	4.09

## Data Availability

Not applicable.
